# A probabilistic method for leveraging functional annotations to enhance estimation of the temporal order of pathway mutations during carcinogenesis

**DOI:** 10.1186/s12859-019-3218-2

**Published:** 2019-12-02

**Authors:** Menghan Wang, Tianxin Yu, Jinpeng Liu, Li Chen, Arnold J. Stromberg, John L. Villano, Susanne M. Arnold, Chunming Liu, Chi Wang

**Affiliations:** 1 Department of Statistics, University of Kentucky, Lexington, USA; 2 Department of Molecular & Cellular Biology, Roswell Park Comprehensive Cancer Center, Buffalo, USA; 3 Markey Cancer Center, University of Kentucky, Lexington, USA; 4 Department of Biostatistics, University of Kentucky, Lexington, USA; 5 Department of Internal Medicine, University of Kentucky, Lexington, USA; 6 Department of Molecular & Cellular Biochemistry, University of Kentucky, Lexington, USA

**Keywords:** Carcinogenesis, Somatic mutations, Pathway analysis, Functional annotations

## Abstract

**Background:**

Cancer arises through accumulation of somatically acquired genetic mutations. An important question is to delineate the temporal order of somatic mutations during carcinogenesis, which contributes to better understanding of cancer biology and facilitates identification of new therapeutic targets. Although a number of statistical and computational methods have been proposed to estimate the temporal order of mutations, they do not account for the differences in the functional impacts of mutations and thus are likely to be obscured by the presence of passenger mutations that do not contribute to cancer progression. In addition, many methods infer the order of mutations at the gene level, which have limited power due to the low mutation rate in most genes.

**Results:**

In this paper, we develop a Probabilistic Approach for estimating the Temporal Order of Pathway mutations by leveraging functional Annotations of mutations (PATOPA). PATOPA infers the order of mutations at the pathway level, wherein it uses a probabilistic method to characterize the likelihood of mutational events from different pathways occurring in a certain order. The functional impact of each mutation is incorporated to weigh more on a mutation that is more integral to tumor development. A maximum likelihood method is used to estimate parameters and infer the probability of one pathway being mutated prior to another. Simulation studies and analysis of whole exome sequencing data from The Cancer Genome Atlas (TCGA) demonstrate that PATOPA is able to accurately estimate the temporal order of pathway mutations and provides new biological insights on carcinogenesis of colorectal and lung cancers.

**Conclusions:**

PATOPA provides a useful tool to estimate temporal order of mutations at the pathway level while leveraging functional annotations of mutations.

## Background

Carcinogenesis is a complex process which involves somatic mutations in a number of key biological pathways and processes. Better understanding the temporal order of somatic mutation occurrences is very important to study the biological mechanism of cancer development and to inform new therapeutic targets. For some cancer types, the temporal order of mutations have been well studied. For example, colorectal cancer is frequently initiated by mutations that affect the Wnt signaling pathway, and then progress upon subsequent mutations in genes involved in MAPK, PI3K, TGF-beta, and p53 signaling pathways [1]. However, for many other cancer types, temporal orders of mutations are still largely unknown.

Large-scale somatic mutation profiling via whole-exome or whole-genome sequencing has provided an unprecedented opportunity for using statistical and computational methods to study carcinogenesis. A number of methods have been developed to infer temporal order of somatic mutations based on cross-sectional genomic sequencing data. One class of methods use a single oncogenetic tree or a mixture of trees to characterize temporal order of mutations [2–4]. A stringent constraint of these methods is that they preclude the possibility of convergence of different paths when different mutations yield the same outcome. A more flexible class of methods [5–11] consider progression networks, which do not assume a tree-like dependency structure among mutations. However, these methods still require full modeling of the dependency structure among mutations. As an alternative, Youn and Simon [12] proposed a probabilistic method to directly estimate the order of mutations without an explicit modeling of the dependency structure.

All the aforementioned methods infer tumor progression at the gene level. However, different patients have mutations in different genes and mutation rates for most genes are very low. Therefore, the power of gene level analysis is usually low. One main reason for this mutational heterogeneity is the mutual exclusivity of gene mutations in a biological pathway [13, 14]. Different patients may have different driver mutations from the same pathway with a converged phenotype of perturbing the pathway. Therefore, studying temporal order of mutations at the pathway level rather than individual gene level is biologically more meaningful. Further, the mutation rate of a pathway is much higher than that of an individual gene so pathway analysis provides a stronger signal on co-occurrence of mutations in different samples, which is the primary information used in tumor progression inference. Because of these advantages, there has been a growing interest to develop methods to perform temporal order analysis at the pathway level [11, 15–17].

A major limitation of current methods is that all mutations are treated equally. A gene or pathway is considered to be functionally altered as long as a non-synonymous mutation occurs in the gene or pathway. However, many non-synonymous mutations are passenger mutations that do not contribute to cancer progression. Failing to control for such noise may lead to spurious results. The impact of passenger mutations may get worse at the pathway level because of an elevated noise level due to the increased number of mutations [16]. One approach for dealing with the noise is to incorporate functional annotation of each mutation in the analysis. Quantification of the functional impact of a mutation has been well studied. Several bioinformatics tools, such as SIFT [18], PolyPhen-2 [19], Mutation Assessor [20], and PROVBEAN [21], have been developed to predict the potential effect of a mutation on the stability and function of human proteins. These prediction tools output a probabilistic score to quantify the likelihood for a mutation to be “functional,” i.e. having an effect on the molecular function causing diseases. Incorporating such information should enhance the accuracy of temporal order analysis of mutations.

In this paper, we propose PATOPA, a probabilistic method to characterize the temporal order of mutations at the pathway level. PATOPA incorporates the functional annotation of each mutation and weigh more on mutations that are likely to be functional. To our knowledge, this is the first attempt to incorporate functional impact into mutation temporal order analysis. Simulation studies are performed to evaluate the accuracy of PATOPA on estimating the temporal order of pathway mutations and to assess the effect of functional impact scores on the estimation. PATOPA has been applied to colorectal and lung whole exome sequencing datasets from TCGA.

## Results

### PATOPA overview

**Fig. 1:**
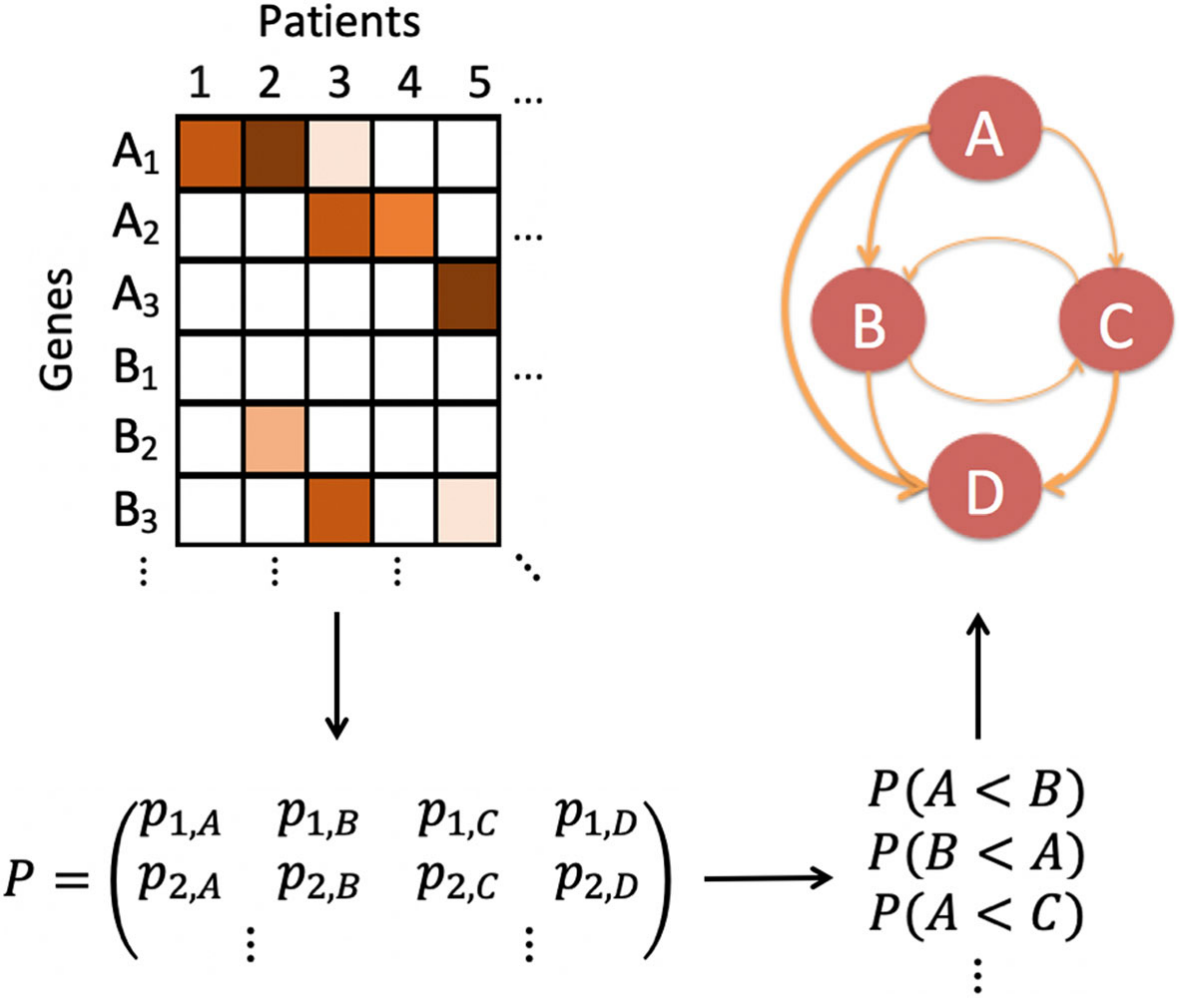
Overview of PATOPA. Suppose we are to determine the temporal order of alterations in four pathways, A, B, C and D. The mutation data for pathways *A*, containing genes *A*_1_, *A*_2_, *A*_3_, and *B*, containing genes *B*_1_, *B*_2_, *B*_3_, are illustrated in the figure using colors, where a darker color indicates a mutation that is more likely to be functional and a lighter color indicates a mutation that is less likely to be functional based on functional annotation. PATOPA integrates the mutation data, pathway information, and functional annotations to estimate a pivotal probability matrix *P*, where the (*k*,*i*) element of the matrix indicates the probability of the *k*th functional mutation occurring in the *i*th pathway. Based on *P*, we infer the temporal order of the four pathways, where *P*(*A*<*B*) is the probability of A being altered before *B* and *P*(*B*<*A*) is the probability of *B* being altered before *A*. Based on those probabilities, a partial order plot is constructed to show the carcinogenesis process, where the thickness of an edge from *A* to *B* is determined by *P*(*A*<*B*)

An overview of PATOPA is provided in Fig. 1. We start from profiles of non-silent somatic mutations along with their associated pathway and functional annotation information for a cohort of patients at various stages of a certain type of cancer. We perform the temporal order analysis at the pathway level instead of individual gene level. A pathway is considered as being functionally altered only if at least one functional mutation has occurred. We use a probabilistic model to estimate a pivotal probability matrix *P* on the ordering of functional mutational events, where the (*k*,*i*) element of the matrix, *p*_*k*,*i*_, indicates the probability of the *k*th functional mutation occurring in the *i*th pathway. Based on this pivotal probability matrix, we calculate the temporal order probability of one pathway being altered before or after another pathway. Finally, we use a partial order plot to summarize the temporal order of all the pathways.

The idea of PATOPA can be illustrated by an example of determining the temporal order of pathways A and B in Fig. 1. Notice that all patients who have mutations in pathway B (patients 2, 3 and 5) also have mutations in pathway A. On the other side, patients 1 and 4 only have mutations in pathway A, but not in pathway B. As we assume driver mutations occur in a sequence, such data would suggest that pathway A is likely to be alterred before pathway B during carcinogenesis. This idea was originated by Youn and Simon [12]. In this paper, we make two major extensions. The first extension is to consider the temporal order at the pathway level instead of gene level, which substantially increases the power. As shown in Fig. 1, the mutation frequency of an individual gene is low. For example, genes *A*_2_ and *B*_3_ only have one mutation each, and the mutations are in different patients. Therefore, there is no sufficient information to confidently determine the temporal order of mutations in these two genes. In contrast, when pooling genes from the same pathway together, the mutation frequency increases substantially for both pathways so that the temporal order estimation becomes more feasible. The second extension is to incorporate functional annotation information to improve the inference. As shown in Fig. 1, patient 5 has mutations in both pathways A and B. If we ignore functional annotation information, data from this patient is not informative to indicate the order of the two pathways. However, based on functional annotation, the mutation in pathway A is likely a functional mutation while the one in pathway B is likely a non-functional mutation. With this added piece of information, data from this patient is useful to support that pathway A is alterred before pathway B.

### Optimization for computational efficiency

**Fig. 2:**
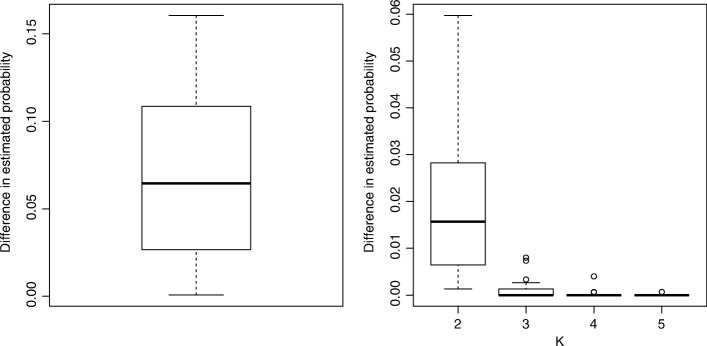
Validity of approaches to improve computational efficacy. Left panel: difference in estimated temporal order probabilities from analyzing each pair of pathways at a time versus all pathways together; right panel: difference in estimated temporal order probabilities between adjacent *K* values, where each boxplot indicates the distribution of difference in estimated probabilities between *K* and *K*−1 across all the 36 pairs of key pathways based on TCGA rectal cancer data

PATOPA involves estimation of the parameter matrix *P*, where the size of the matrix is determined by the number of pathways and the number of functional mutation events. Without control of these two numbers, *P* can contain a large number of parameters, which makes the estimation computationally intensive. We used the following two approaches to reduce number of parameters in *P*. Firstly, we performed our analysis for each pair of pathways seperately to estimate *P* and temporal order probabilities. This approach substantially reduces the number of columns in *P*, and therefore is computationally much more efficient. To examine the performance of this approach, we used TCGA rectal cancer data and analyzed 9 key cancer pathways from the Kyoto Encyclopedia of Genes and Genome (KEGG) database [22]. A more detailed description of the dataset and pathway information is provided in the “Real data analysis” section. The left panel of Fig. 2 compares the estimated temporal order probabilities from analyzing each pair of pathways at a time versus all pathways together. The differences were small with most of the values less than 0.1. Therefore, we used this pairwise analysis approach in the rest of the paper.

Secondly, we set *p*_*k*,*i*_=*p*_*K*,*i*_ for *k*>*K* and estimated an averaged distribution for mutations occurring after the *K*-th step. To choose an appropriate *K* value, we again used the TCGA rectal cancer data and compared estimated temporal order probabilities between adjacent *K* values for 36 pairs of the 9 key pathways. Figure 2 right panel shows that the estimated temporal order probabilities became very stable for *K*≥4. In addition, for each pair of pathways, we calculated the probability distribution of number of functional mutations they would contain (see Additional file 1: Figure S10). The probability of having more than 4 functional mutations for most pairs of pathways was very small. Therefore, we set *K*=4 in our subsequent analyses.

### Simulation studies

#### Evaluating the estimation accuracy of the temporal order of pathway mutations

Simulation studies were conducted to evaluate the performance of PATOPA in determining the temporal order of two pathways, A and B. Our goal was to use simulated datasets that we knew the true pathway order probabilities to investigate whether PATOPA was able to uncover those probabilities when analyzing the datasets. To mimic real world situation, we set the true pathway order probabilities based on TCGA rectal cancer mutation data from the p53 signaling (our pathway A, 8 genes) and cell cycle (our pathway B, 89 genes) pathways. Specifically, we applied PATOPA to TCGA rectal cancer data to estimate *p*_*k*,*A*_ and *p*_*k*,*B*_, the probability that the *k*th functional mutation was from pathways A and B, respectively. We also calculated the probabilities of pathway *A* being altered before (*P*(*A*<*B*)), simultaneously with (*P*(*A*=*B*)), and after (*P*(*A*>*B*)) pathway *B* being altered. These probability values were set as true values to simulate data based on the following procedure. Firstly, the number of functional and non-functional mutations in a patient were generated based on the empirical distributions in TCGA rectal cancer data, respectively. Secondly, functional mutations were assigned to pathways in a temporal order, where the *k*th functional mutation was assigned to pathway A with probability *p*_*k*,*A*_, or to pathway B with probability *p*_*k*,*B*_. Thirdly, non-functional mutations were randomly assigned to the two pathways with probabilities *q*_*i*_, the probability that a randomly sampled non-functional mutation is from pathway *i* for *i*=*A* or *B*. Fourthly, the functional impact score of each gene mutation was assigned as the conditional probability of observing this specific mutation given that there was a functional/non-functional mutation in the pathway that this gene belonged to.

We simulated data of sample size 50, 100, 200 or 400 with 100 replicates at each sample size. We applied PATOPA to simulated datasets to estimate *P*(*A*<*B*), *P*(*A*=*B*) and *P*(*A*>*B*). To quantify the difference between true probability values we set when simulating the data and estimated probability values from PATOPA, we define the bias as the mean absolute difference between true and estimated probability values across 100 simulations. As shown in Table 1, the bias was small, indicating that PATOPA was able to accurately estimate those probabilities. In addition, the bias decreased as the sample size increased, indicating that PATOPA had increased precision in estimating those probabilities when more data were available.

**Table 1:** Estimation accuracy of PATOPA. Numbers presented are the bias, i.e. the difference between the estimated and true values averaged across 100 simulation replicates

Sample size	*P*(*A*>*B*)	*P*(*A*=*B*)	*P*(*A*<*B*)
50	0.074	0.069	0.041
100	0.048	0.045	0.028
200	0.038	0.034	0.021
400	0.021	0.020	0.013

#### Evaluating the effect of functional impact scores

**Fig. 3:**
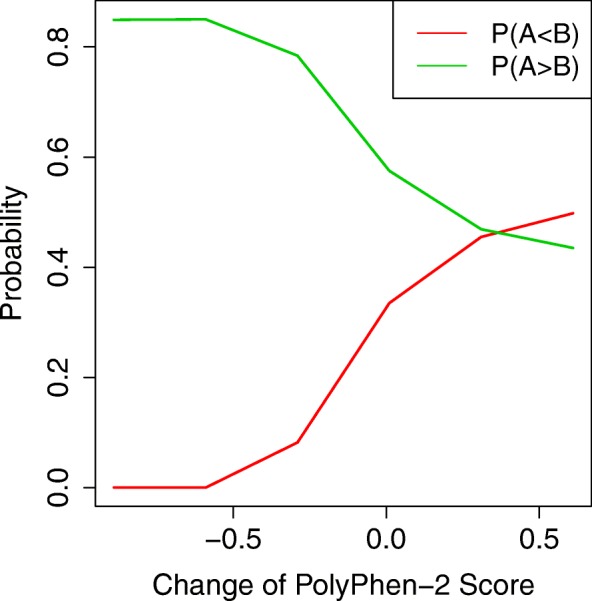
Effect of functional impact scores on the inference of temporal order of pathway mutations. PolyPhen-2 scores of all mutations in pathway A were changed by a certain amount and the corresponding estimated temporal order probabilities were presented

We performed another set of simulations to assess the effect of functional impact score on the estimation of temporal order probabilities. Under the same simulation setting described in the previous subsection, we increased or decreased the PolyPhen-2 scores of all mutations in the p53 signaling pathway (pathway A). The resulting probabilities are presented in Fig. 3. It shows the trend that when PolyPhen-2 scores of mutations in pathway A were decreased, *P*(*A*<*B*) decreased and *P*(*A*>*B*) increased. Specifically, when functional impact scores of all pathway A mutations were decreased by 0.5, *P*(*A*<*B*) decreased to 0.25. When scores were increased by 0.5, *P*(*A*<*B*) increased to 0.7. The results demonstrate the substantial impact of functional impact score that can lead to distinct inference on the temporal order of pathways.

### Real data analysis

To further evaluate the performance of PATOPA, we applied it to TCGA whole exome sequencing data for colorectal and lung cancer data. We considered 9 key cancer pathways in our analysis. In addition to the canonical molecular signaling pathways of wingless-related integration site (WNT), mitogen-activated protein kinase (MAPK), phosphoinositide 3-kinase (PI3K), transforming growth factor beta (TGF-beta), p53 and vascular endothelial growth factor (VEGF), we also included pathways involving the processes of apoptosis, adherens junction and cell cycle. The apoptosis pathway is an indicator of turnover of both normal and tumor cells; the adherens junction pathway is an important factor for tumor invasion; and the cell cycle pathway suggests the process of cell growth in the tumor. Genes in each of the 9 pathways, which are listed in Additional file 1: Table S1, were determined based on the KEGG database [22] with manual curation.

#### Analysis of TCGA colorectal cancer data

**Fig. 4:**
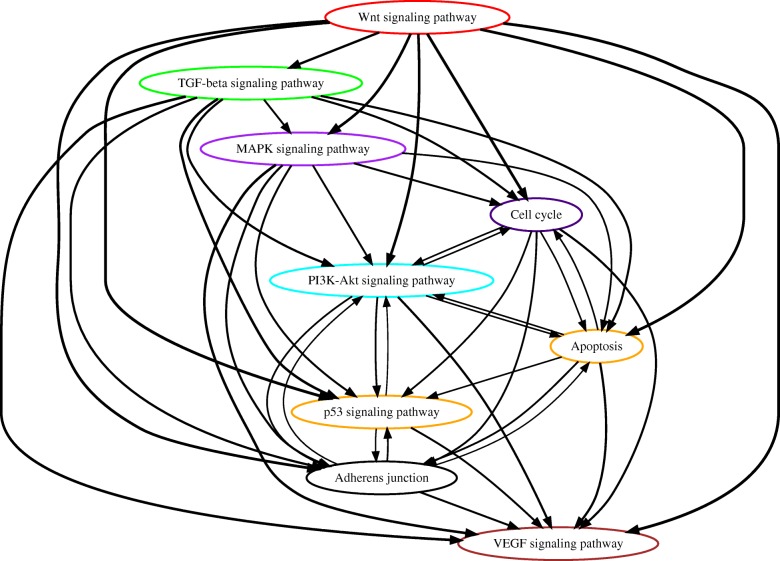
Estimated order of pathway alterations based on TCGA rectal cancer dataset. Estimated temporal order probabilities from PATOPA are summarized using a partial order plot [24]. Each node corresponds to a pathway we analyzed. The nodes/pathways are ordered based on the temporal order probabilities estimated from PATOPA. The thickness of a directed edge is proportional to the probability that the head node is mutated after the tail node. Pathways with similar temporal order probabilities are clustered together, where the clustering results are indicated by different colors of node borders. For better visualization, edges with probability less than 0.4 are removed from this figure

**Fig. 5:**
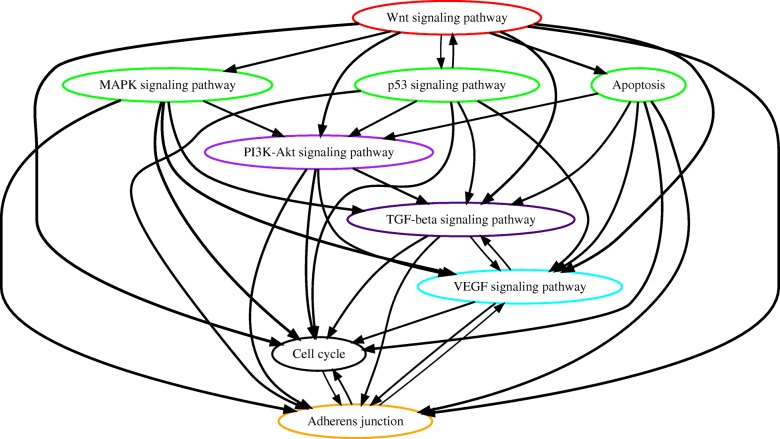
Estimated order of pathway alterations based on TCGA colon cancer dataset. Estimated temporal order probabilities from PATOPA are summarized using a partial order plot [24]. Each node corresponds to a pathway we analyzed. The nodes/pathways are ordered based on the temporal order probabilities estimated from PATOPA. The thickness of a directed edge is proportional to the probability that the head node is mutated after the tail node. Pathways with similar temporal order probabilities are clustered together, where the clustering results are indicated by different colors of node borders. For better visualization, edges with probability less than 0.4 are removed from this figure

The TCGA project provided the whole exome sequencing data of 461 colon and 172 rectal tumor samples. Since we aimed to better understand the similarity and difference of carcinogenesis between colon and rectal cancers, we analyzed these two datasets separately. For each cancer type, we deleted the top 16% hyper-mutated samples because the carcinogenesis process of these tumors involve different sequences of genetic events [23]. We applied PATOPA to estimate temporal order probabilities of each pair of pathways and the results are summarized by partial order plots (Figs. 4 and 5). The comparison between the orders PATOPA found from seperate analysis of rectal cancer and colon cancer mutation data and those reported in the literature for colorectal combined tumor [1] is presented in Figure S11 in Additional file 1. Most of the inferred temporal orders of pathway mutations were consistent with cancer research literature. Specifically, the estimated temporal orders of WNT - MAPK - PI3K - p53 signaling pathways for rectal cancer and WNT - MAPK - PI3K - TGF-beta signaling pathways for colon cancer were the same as the known sequences of biological events in colorectal cancer [1]. Interestingly, the TGF-beta pathway were placed before the MAPK pathway from our analysis of rectal cancer alone (Fig. 4), and the p53 signaling pathway was placed before the PI3K and TGF-beta signaling pathways from our analysis of colon cancer alone (Fig. 5), which are cancer type-specific and distinct from the biological evidence for “colorectal” combined tumor [1]. This might be due to the lack of biological evidence from the isolated rectal and colon cancer samples separately and the traditional method for tissue collection and analysis from colorectal cancer patients. Also, traditional biological analysis only considered very limited number of gene mutations in each pathway, while PATOPA analysis considered all of the available mutations in each defined pathway.

To better illustrate how the incorporation of functional impact scores benefited our analysis, we studied the distribution of PolyPhen-2 scores for each pathway using TCGA rectal and colon cancer datasets, see Figure S12 in Additional file 1. The Wnt signaling pathway had more mutations with high PolyPhen-2 scores than the MAPK and p53 signaling pathways, which supports our model inference that the Wnt signaling pathway was altered prior to the MAPK and p53 signaling pathways. Our model is based on the idea that only mutations affecting protein functions should be used to infer mutational order of pathways. Since the PolyPhen-2 score quantifies the probability of each mutation being functional, pathways having more mutations with high PolyPhen-2 scores tend to be ordered earlier than pathways having fewer such mutations.

#### Analysis of TCGA lung cancer data

**Fig. 6:**
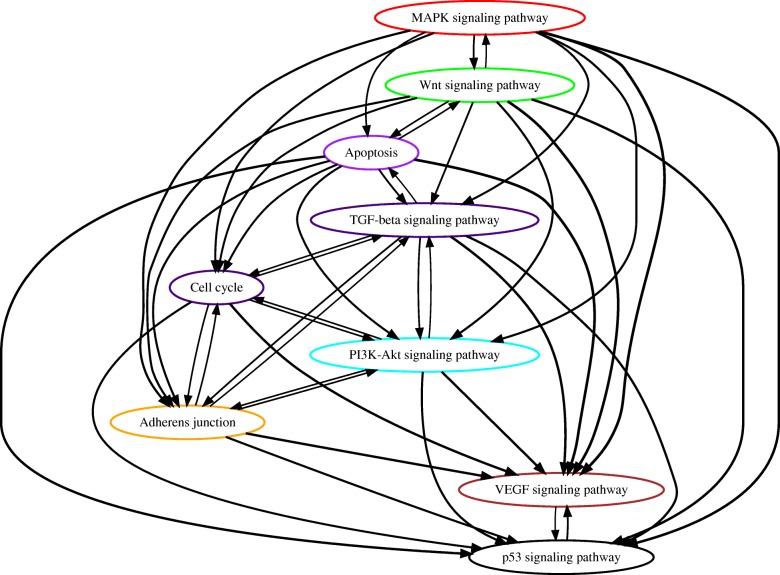
Estimated order of pathway alterations based on TCGA lung adenocarcinoma dataset. Estimated temporal order probabilities from PATOPA are summarized using a partial order plot [24]. Each node corresponds to a pathway we analyzed. The nodes/pathways are ordered based on the temporal order probabilities estimated from PATOPA. The thickness of a directed edge is proportional to the probability that the head node is mutated after the tail node. Pathways with similar temporal order probabilities are clustered together, where the clustering results are indicated by different colors of node borders. For better visualization, edges with probability less than 0.4 are removed from this figure

**Fig. 7:**
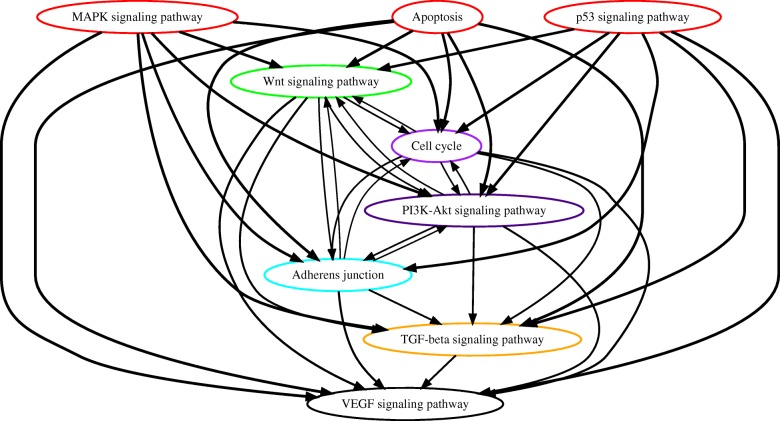
Estimated order of pathway alterations based on TCGA lung squamous cell carcinoma dataset. Estimated temporal order probabilities from PATOPA are summarized using a partial order plot [24]. Each node corresponds to a pathway we analyzed. The nodes/pathways are ordered based on the temporal order probabilities estimated from PATOPA. The thickness of a directed edge is proportional to the probability that the head node is mutated after the tail node. Pathways with similar temporal order probabilities are clustered together, where the clustering results are indicated by different colors of node borders. For better visualization, edges with probability less than 0.4 are removed from this figure

Non-small cell lung cancer is another significant cancer type to our interest, which is primarily composed of two clinically and pathologically different subtype groups, i.e. lung adenocarcinoma and lung squamous cell carcinoma. We applied PATOPA to the whole-exome sequencing data of 585 lung adenocarcinoma samples and 504 lung squamous cell carcinoma samples in TCGA. The resulting partial order plots are shown in Fig. 6 and Fig. 7. The mutations in the MAPK signaling pathway ranked on the top of lung adenocarcinoma. This is not surprising as KRAS is the most frequently mutated gene in the lung adenocarcinoma but less frequent in the lung squamous cell carcinoma [25, 26]. In both lung adenocarcinoma and squamous cell carcinoma, the mutations of the Wnt signaling ranked just below the MAPK signaling. It has been reported that activation of both Wnt signaling and KRAS dramatically enhanced lung carcinogenesis [27]. However, from biological evidences, the most prevalent mutations found in lung cancer are those of p53 signaling pathway. Interestingly, we notice that mutation of the p53 pathway appeared to be in distinct positions of orders in lung adenocarcinoma and squamous cell carcinoma. While p53 pathway mutation was downstream of most of the other pathway mutations in lung adenocarcinoma, it was at the upstream of all signaling pathway mutations in lung squamous cell carcinoma (Fig. 6 and Fig. 7). Previous findings suggest that p53 pathway mutations are involved in 80% of lung squamous cell carcinoma, while the mutations are involved in 50% of lung adenocarcinoma [28]. Our model explains the possibility that in lung squamous cell carcinoma, p53 pathway plays a more fundamental role in initiating the tumor cell growth than in lung adenocarcinoma.

#### Comparison to other method

We compared PATOPA with an existing method, H-CBN [15]. We focused our comparison on colorectal cancer, for which the temporal order of pathway alterations is better understood in cancer research literature. We applied H-CBN to TCGA rectal and colon data. The obtained pathway order estimates are provided in Additional file 1: Figure S13. For rectal cancer, H-CBN was able to infer that Wnt pathway was alterred before MAPK pathway, and MAPK pathway was alterred before PI3K-Akt pathway. However, it was unable to determine the orders of Wnt/MAPK/PI3K and p53 and TGF-beta pathways. Therefore, temporal orders inferred by PATOPA were closer to the cancer research literature than those inferred by H-CBN. For colon cancer, H-CBN was able to infer the orders of MAPK and PI3K pathways, and MAPK and p53 pathways. However, H-CBN was unable to determine the order of MAPK/PI3K/p53 and Wnt and TGF-beta pathways. Therefore, the results from H-CBN were less informative that those from PATOPA.

## Discussion

Inferring temporal order of driver mutations during carcinogenesis is an important task in the analysis of whole genome/exome sequencing data. Considering mutations at the pathway level rather than individual gene level is biologically meaningful and can substantially increase the power of the analysis. We focused on 9 KEGG cancer-related pathways in our data analysis. But our method is generally applicable to study alterations of other biological pathways or gene sets including de novo driver gene sets identified from computational algorithms [14, 29].

In our analysis, we used PolyPhen-2 score [19] to characterize the probability of each mutation being functional. Other scores, such as SIFT [18], Mutation Assessor [20] and PROVBEAN [21], can also be used alternatively. One limitation of our analysis is that we only considered single nucleotide variants and did not include copy number variants. Incorporating copy number variants into the analysis is our future work.

Our data analysis did not account for cancer subtypes [30–33]. It would be interesting to perform the analysis within each cancer subtype separately and compare the temporal order of pathway alterations between different subtypes. Such analysis may identify subtype-specific pathway alteration orders and better understand the development process of a certain cancer subtype, although the sample size may be a limiting factor of the power of the analysis.

PATOPA infers temporal order of pathway mutations based on mutation frequencies across a cohort of patients. It does not account for the intra-tumor heterogeneity [34, 35], which may limit the method’s ability to discern temporal order of pathway mutations in some cases. Recent advances in bioinformatic tools enables us to reconstruct the evolutionary history and population frequency of the subclonal lineages of tumor cells based on single- or multi-region sequencing of samples from an individual patient [36–38]. In addition, emerging single-cell sequencing technologies [39–41] have the promise of revealing tumor heterogeneity at a much higher resolution. Tumor evolutionary lineage can be reconstructed [42, 43] based on single-cell sequencing data. Incorporating intra-tumor heterogeneity and tumor evolution information may substantially improve the estimation of pathway mutation orders, which is an important direction of future research.

## Conclusions

In this article, we have proposed PATOPA, a new probabilistic method for inferring the temporal order of pathway mutations during carcinogenesis based on whole genome/exome sequencing data and functional impact scores of mutations. The method can be a useful tool to help researchers better understand the process of tumor development. The result obtained by applying our method to TCGA rectal cancer whole-exome sequencing data is mostly consistent with the multi-step process of colorectal carcinogenesis established by previous research, which provides a degree of validation of the ability of our method to recover mutation order of pathways from a cross-sectional dataset.

## Methods

### A probabilistic approach

Our goal is to determine the order of *N* pathways. We first consider the case that all the pathways do not have any gene in overlap. An extension of our method to the case of overlapping pathways is provided at the end of this subsection. Let $Y^{j}_{i}$ be the observed number of non-synonymous mutations in pathway *i* of patient j for *i*=1,…,*N*, *j*=1,…,*M*, and $m_{j} = \sum _{i=1}^{N} Y_{i}^{j}$ be the total number of non-silent mutations in patient *j*. Let $S_{k}^{j}$ indicate whether the *k*th mutation is functional ($S_{k}^{j}$=1) or not ($S_{k}^{j}$=0) for *k*=1,…,*m*_*j*_, and $n_{j}=\sum _{k=1}^{m_{j}} S_{k}^{j}$ be the total number of functional mutations in patient *j*. Based on the law of total probability, the probability of observing the set of $Y^{j}_{i}$ can be expressed as

1 $$ \begin{aligned} &P(Y^{j}_{1}, \ldots, Y^{j}_{N})\\ =& \Sigma \left. P(Y^{j}_{1}, \ldots, Y^{j}_{N}|S_{1}^{j},...,S_{m_{j}}^{j},m_{j}) P(S_{1}^{j},...,S_{m_{j}}^{j} | m_{j})P(m_{j}),\right. \end{aligned}  $$

where the summation is over all possible sequences of $S_{1}^{j},...S_{m_{j}}^{j}$. We next describe how to calculate each of the three terms in the summation. For the first term, let $D^{j}_{k}$ denote the unknown identity of the pathway mutated as the *k*th functional event, and $N^{j}_{v}$ denote the unknown identity of the pathway mutated as the *v*th nondamaging event in patient *j*. We have

2 $$ {\begin{aligned}  &  P(Y^{j}_{1}, \ldots, Y^{j}_{N}|S_{1}^{j},...S_{m_{j}}^{j},m_{j}) \\ = \Sigma &P(D^{j}_{1}=i_{1}, \ldots, D^{j}_{n_{j}}=i_{n_{j}},N^{j}_{1}=i_{n_{j}+1}, \ldots,N^{j}_{m_{j}-n_{j}}=i_{m_{j}} |S_{1}^{j}, \\ &\ldots, S_{m_{j}}^{j}, m_{j}) \\ =\Sigma &P(D^{j}_{1}=i_{1}, \ldots, D^{j}_{n_{j}}=i_{n_{j}}|S_{1}^{j}, \ldots, S_{m_{j}}^{j}, m_{j}) \\ &\times P(N^{j}_{1}=i_{n_{j}+1}, \ldots,N^{j}_{m_{j}-n_{j}}=i_{m_{j}} |S_{1}^{j}, \ldots, S_{m_{j}}^{j}, m_{j}), \end{aligned}}  $$

where the summation is over all possible orders of pathway identities of functional and non-functional mutations, $\phantom {\dot {i}\!}(i_{1}, \ldots, i_{m_{j}})$, that are consistent with the observed set of mutations $(Y^{j}_{1}, \ldots, Y^{j}_{N})$, and the last equation is obtained by assuming the occurrences of functional mutations and non-functional mutations are independent. For functional mutations, let $p_{k,i_{k}}$ be the probability that the *k*th functional mutation occurs in the *i*_*k*_th pathway and assume that $p_{k,i_{k}}$ is independent of $p_{l,i_{l}}$ for *l*≠*k*. The $p_{k,i_{k}}$’s are our parameters of interest. For non-functional mutations, as their orderings are most likely random, we assume equal probablity for each order of non-functional mutations in a given patient. Let *q*_*i*_ be the probability that a randomly sampled non-functional mutation is from pathway *i*, equation (2) can be re-written as

$$\Sigma \left(\prod \limits^{n_{j}}_{k=1} p_{k, i_{k}} \right) \left(\prod\limits_{l=n_{j}+1}^{m_{j}} q_{i_{l}} \right). $$

In practice, we estimate *q*_*i*_ by an average across samples, i.e. $ (\sum _{j} E_{i}^{j})/(\sum _{j} \sum _{z} E_{z}^{j}) $, where $E_{z}^{j} = \sum _{k: \textrm {the }k\textrm {th mutation is from pathway }z} (1-r_{k})$ is the expected number of non-functional mutations in pathway *z* for patient *j* and $r_{k}=P(S_{k}^{j}=1)$ is the functional impact score of the *k*th mutation that can be obtained from software such as PolyPhen-2 [19].

For the second term in equation (1), we assume $S_{k}^{j}$ is independent of $S_{l}^{j}$ for *l*≠*k*. We have $P(S_{1}^{j},...,S_{m_{j}}^{j} | m_{j}) = \prod \limits ^{m_{j}}_{k=1} r_{k}^{S_{k}^{j}}(1-r_{k})^{1-S_{k}^{j}}$. For the third term in equation (1), since the marginal probability *P*(*m*_*j*_) is independent of $p_{k,i_{k}}$, it can be ignored in the likelihood function. Therefore, the likelihood function can be written as

3 $$ {\begin{aligned}  \prod \limits_{j} \Sigma \left\{\Sigma \left(\prod \limits^{n_{j}}_{k=1} p_{k,i_{k}} \right) \left(\prod\limits_{l=n_{j}+1}^{m_{j}} q_{i_{l}} \right) \right\} \left\{\prod \limits^{m_{j}}_{k=1} r_{k}^{S_{k}^{j}}(1-r_{k})^{1-S_{k}^{j}}\right\}. \end{aligned}}  $$

An estimate of $p_{k,i_{k}}$ is obtained by maximizing the likelihood, see the “Parameter estimation” section. Finally, the probability of pathway *A* being alterred prior to pathway *B*, denoted by *P*(*A*<*B*), is

$${} P(A\! < B) = \Sigma_{\{(i_{1}, \ldots, i_{n}) \in G_{A<B}\}} \left(\prod \limits^{n}_{k=1} p_{k,i_{k}}\right), n = {\underset{j \in \{1,\ldots,m\}}{\max}} \{m_{j}\} $$

where *G*_*A*<*B*_ is the subset of pathway mutation sequences satisfying that the first functional mutation in *A* occurs before the first functional mutation in *B* occurs.

The aforementioned method requires that the pathways have no overlap. However, many pathways in biological databases, such as KEGG [22], have overlapped genes. In such situation, we regroup the genes into mutually exclusive gene sets to run the analysis. Consider an example of two pathways, *A* and *B*, with an overlapped subset *A*∩*B*. We regroup the genes into three mutually exclusive sets: *A*^′^=*A*∩*B*^*c*^, *AB*=*A*∩*B*, and *B*^′^=*A*^*c*^∩*B*, and perform the analysis on those three sets. Functional mutations in *A*^′^ and *B*^′^ are able to delineate the temporal order of *A* and *B*. Functional mutations in *AB* are considered as altering both *A* and *B* simultaneously and are used to estimate such probability, i.e. *P*(*A*=*B*).

### Visualization

We visualize the result of our analysis of each cancer type with a partial order plot [24]. Each nodes in the plot corresponds to a pathway. Nodes are ordered based on estimated temporal order probabilities using the layered graph drawing method in Graphviz (version 1.3.1) [44], where pathways likely to be mutated at early stage are placed on the top while pathways likely to be mutated at late stage are placed at the bottom. The thickness of a directed edge is proportional to the probability that the head node (pathway) is mutated before the tail node (pathway). For better visualization, edges with probabilities less than a threshold value are removed from the plot. In addition, we cluster pathways using the correlation clustering algorithm [45], which aims to find a clustering that simultaneously maximizes the similarities (the probability that the order of two pathways cannot be determined) between clusters and minimizes the dissimilarities (the probabilities that the order of the two pathways can be determined) between clusters. The clustering results are presented by colors of the node borders.

### Parameter estimation

The estimator of *p*_*k*,*i*_ is obtained by maximizing the likelihood function (3). Since the *p*_*k*,*i*_’s need to satisfy the constraints 0≤*p*_*k*,*i*_≤1 and $\Sigma _{i=1}^{N} p_{k,i}=1$, we consider the following parameter transformation:

$$\begin{array}{*{20}l} p_{k,i_{k}}=&\frac{\exp(\omega_{k,i_{k}})}{\sum \limits^{m_{j}-1}_{l=1}\exp(\omega_{l,i_{l}})+1},~k=1,...,m_{j}-1 \textrm{ and } p_{m_{j},i_{m_{j}}}\\=&\frac{1}{\sum \limits^{m_{j}-1}_{l=1}\exp(\omega_{l,i_{l}})+1} \end{array} $$

where $\omega _{k,i_{k}}$’s are unconstrained parameters whose estimates are obtained by the Nelder and Mead method.

### Pathway definition

We consider 9 key cancer pathways from the KEGG database [22] in our real data analysis. To minimize overlaps between pathways, the pathway genes connected by “O or e” (transcriptional regulation), “- - -” (indirect regulation) or " ||” (cell membrane) are separated. Only the “core” pathway genes are selected. For the apoptosis pathway, PI3K and RAS were excluded but TP53 was included. This is because PI3K and RAS pathway regulate the transcription of apoptosis genes, while TP53 not only regulates transcription, but also has transcription-independent function in apoptosis. For the PI3K-Akt signaling pathway, all genes downstream of AKT were excluded because they belong to other pathways that are defined as independent pathways in the KEGG database. The defined gene sets for each pathway are listed in Table S1 in Additional file 2 and displayed in Figures S1-S9 in Additional file 1.

## Availability and requirements

**Project name:** PATOPA**Project home page:**https://github.com/MarkeyBBSRF/PATOPA.**Operating system(s):** Linux or Windows**Programming language:** R, C**Other requirements:** R 3.3.2, gcc 4.2.1**License:** GNU GPL**Any restrictions to use by non-academics:** None

## Supplementary information

**Additional file 1** Figures S1-S9 display the “core” pathway genes in each of the 9 key cancer pathways used in the paper. Figure S10 shows the distribution of the number of functional mutations in pairs of pathways. Figure S11 compares the temporal orders of pathway alterations inferred by PATOPA with the literature. Figure S12 shows the distribution of PolyPhen-2 scores in each pathway. Figure S13 shows the inferred temporal order of pathway mutations from TCGA rectal and colon data based on H-CBN.

**Additional file 2** Table S1 provides a list of “core” pathway genes in each of the 9 key cancer pathways considered in the paper.

## Data Availability

PATOPA is available at https://github.com/MarkeyBBSRF/PATOPA.

## Abbreviations

TCGA: The Cancer Genome Atlas
KEGG: Kyoto Encyclopedia of Genes and Genomes
Wnt: Wingless-related integration site
MAPK: Mitogen-activated protein kinase
PI3K: Phosphoinositide 3-kinase
TGF-beta: Transforming growth factor beta
VEGF: Vascular endothelial growth factor
