# Supplementary material for: A probabilistic method for leveraging functional annotations to enhance estimation of the temporal order of pathway mutations during carcinogenesis
Source: BMC Bioinformatics. 2019 Dec 2;20:620. doi: 10.1186/s12859-019-3218-2 (PMC6889196; doi:10.1186/s12859-019-3218-2)
Supplement: Supplementary file 1 — Additional file 1 Figures S1-S9 display the “core” pathway genes in each of the 9 key cancer pathways used in the paper. Figure S10 shows the distribution of the number of functional mutations in pairs of pathways. Figure S11 compares the temporal orders of pathway alterations inferred by PATOPA with the literature. Figure S12 shows the distribution of PolyPhen-2 scores in each pathway. Figure S13 shows the inferred temporal order of pathway mutations from TCGA rectal and colon data based on H-CBN. [file 12859_2019_3218_MOESM1_ESM.pdf]

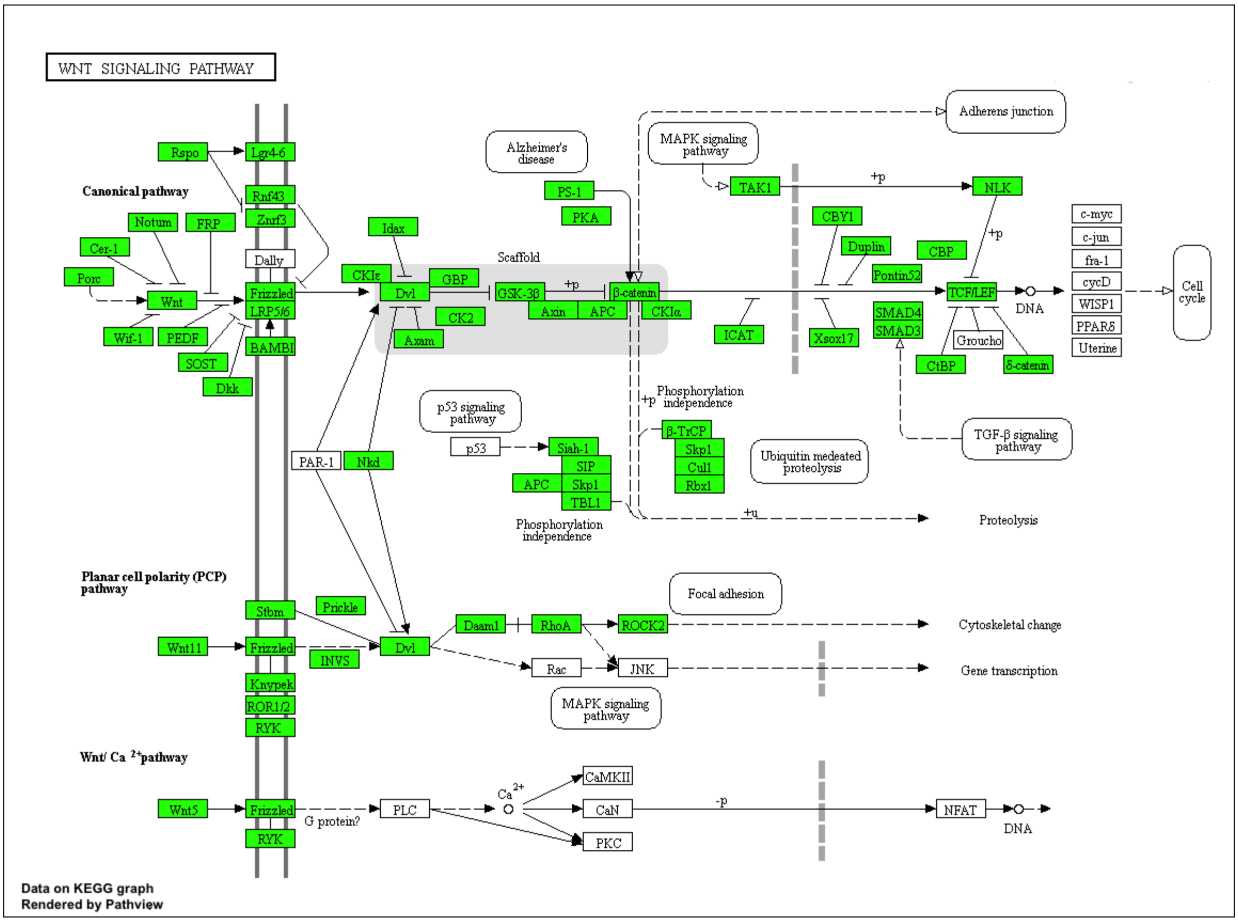

FIG. S1: **The Wnt signaling pathway.** Based on the KEGG Wnt signaling pathway (hsa04310), we further selected "core" pathway genes as described in the pathway definition subsection of the main text. Our analysis only used the "core" pathway genes, which are hightlighted in green. The figure was generated based on Pathview [4].

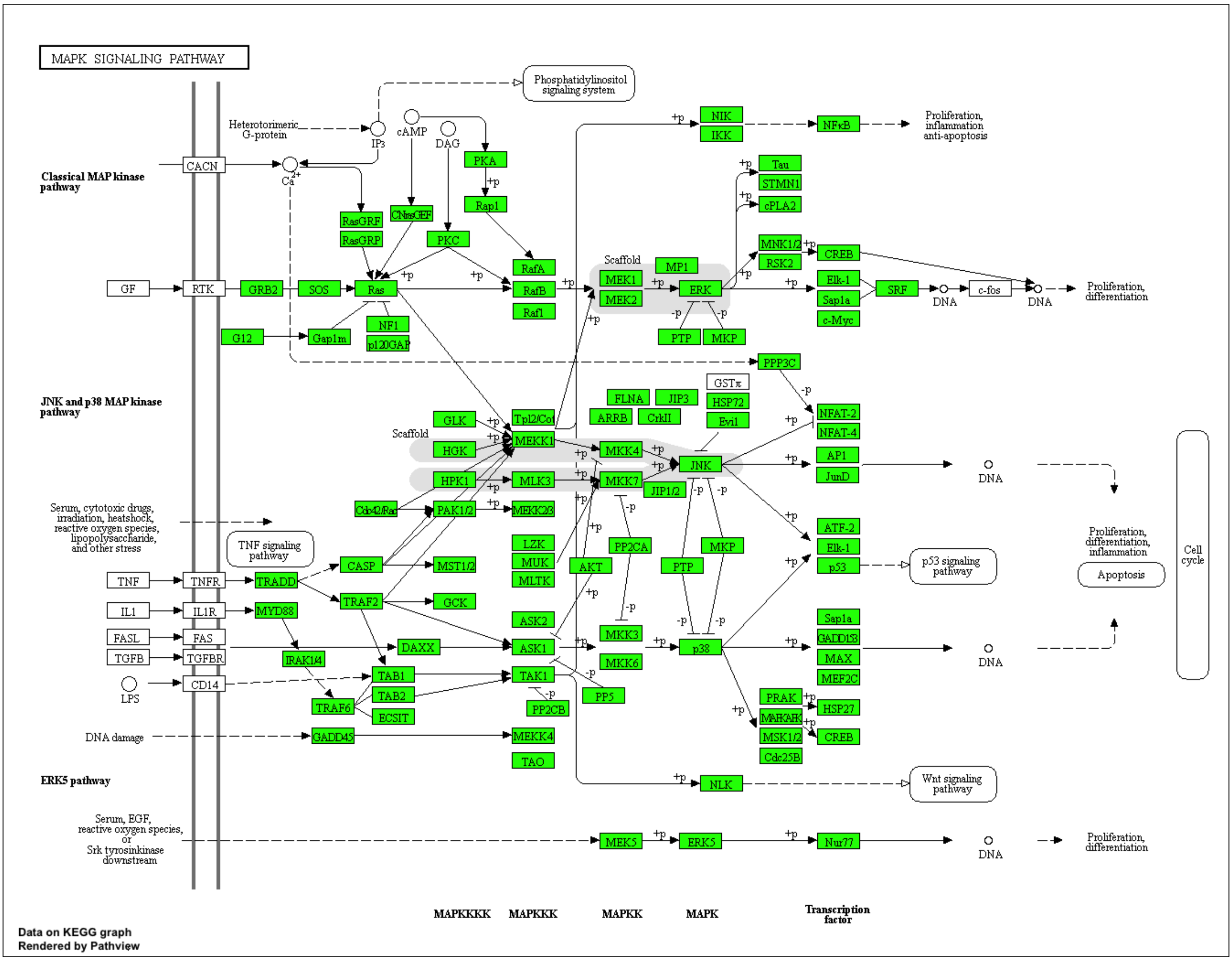

FIG. S2: **The MAPK signaling pathway.** Based on the KEGG MAPK signaling pathway (hsa04010), we further selected "core" pathway genes as described in the pathway definition subsection of the main text. Our analysis only used the "core" pathway genes, which are hightlighted in green. The figure was generated based on Pathview [4].

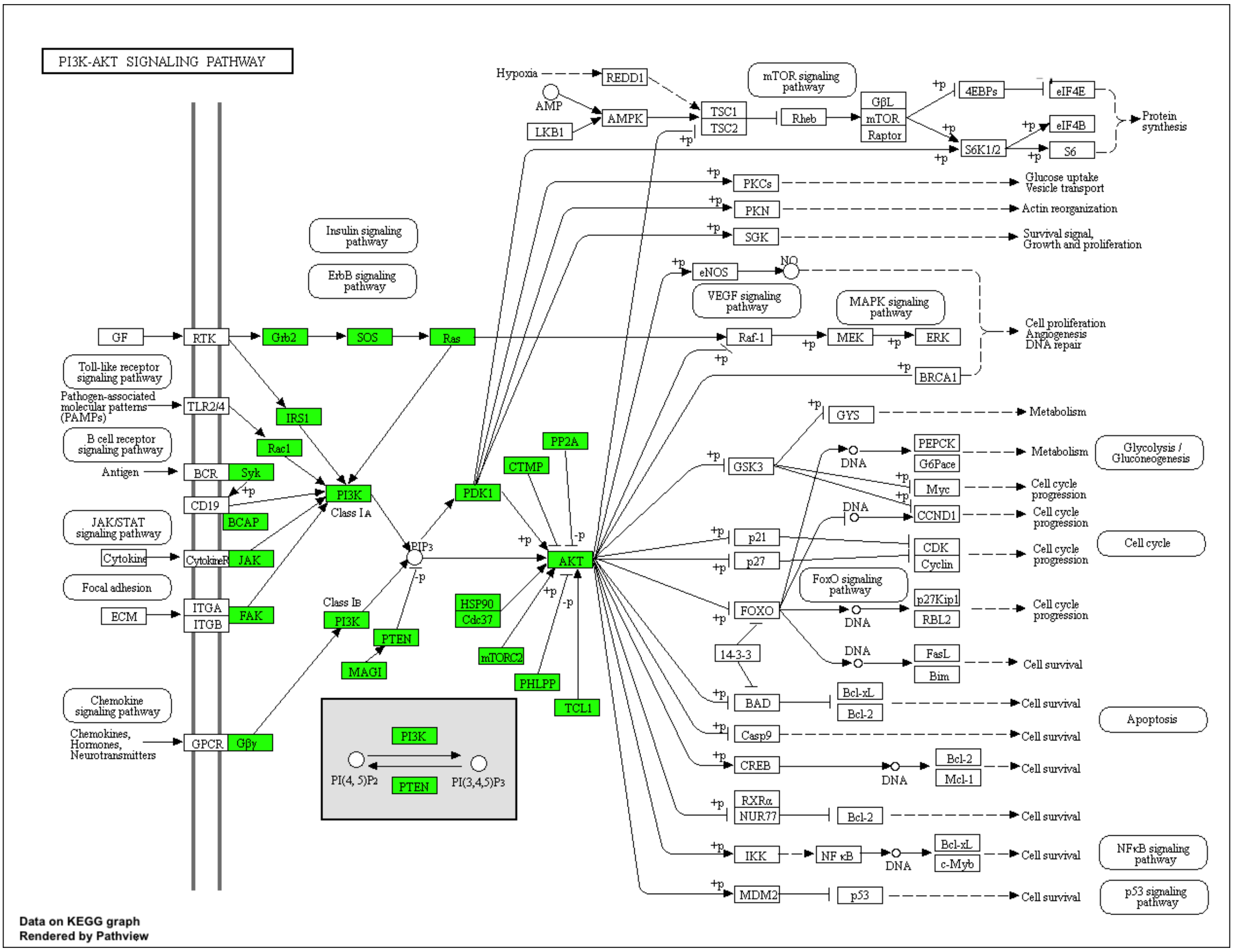

FIG. S3: **The PI3K signaling pathway.** Based on the KEGG PI3K signaling pathway (hsa04151), we further selected "core" pathway genes as described in the pathway definition subsection of the main text. Our analysis only used the "core" pathway genes, which are hightlighted in green. The figure was generated based on Pathview [4].

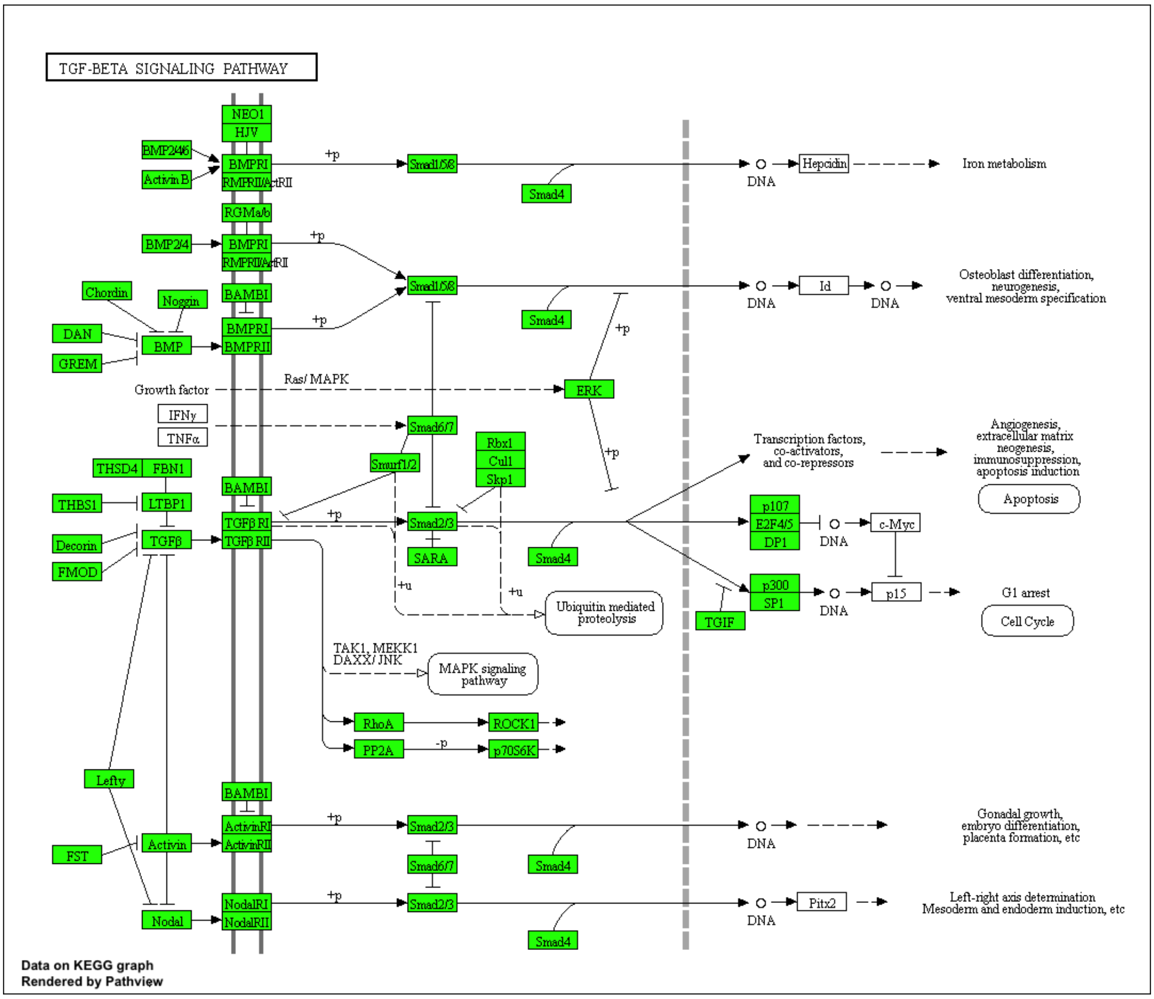

FIG. S4: **The TGF-beta signaling pathway.** Based on the KEGG TGF-beta signaling pathway (hsa04350), we further selected "core" pathway genes as described in the pathway definition subsection of the main text. Our analysis only used the "core" pathway genes, which are hightlighted in green. The figure was generated based on Pathview [4].

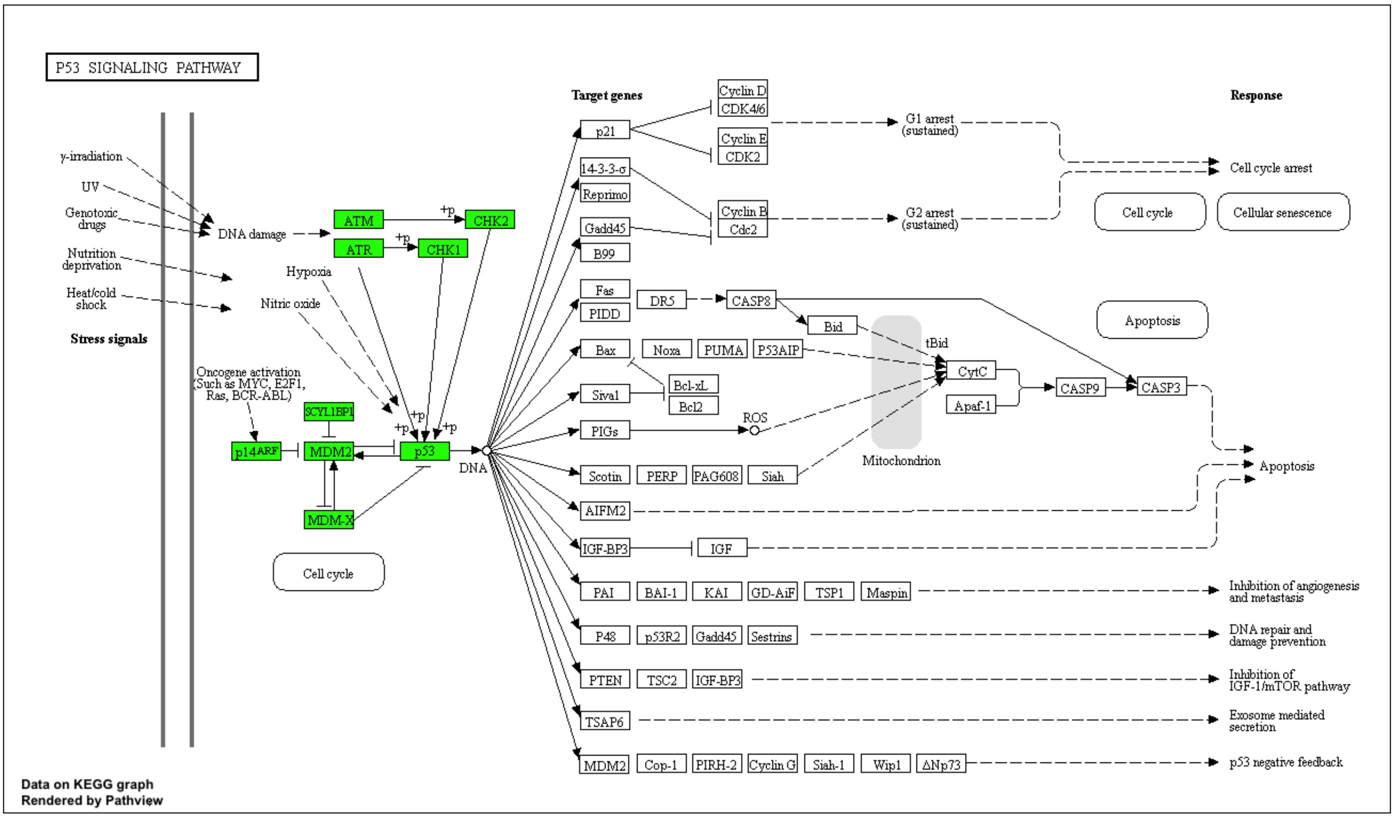

FIG. S5: **The p53 signaling pathway.** Based on the KEGG p53 signaling pathway (hsa04115), we further selected "core" pathway genes as described in the pathway definition subsection of the main text. Our analysis only used the "core" pathway genes, which are hightlighted in green. The figure was generated based on Pathview [4].

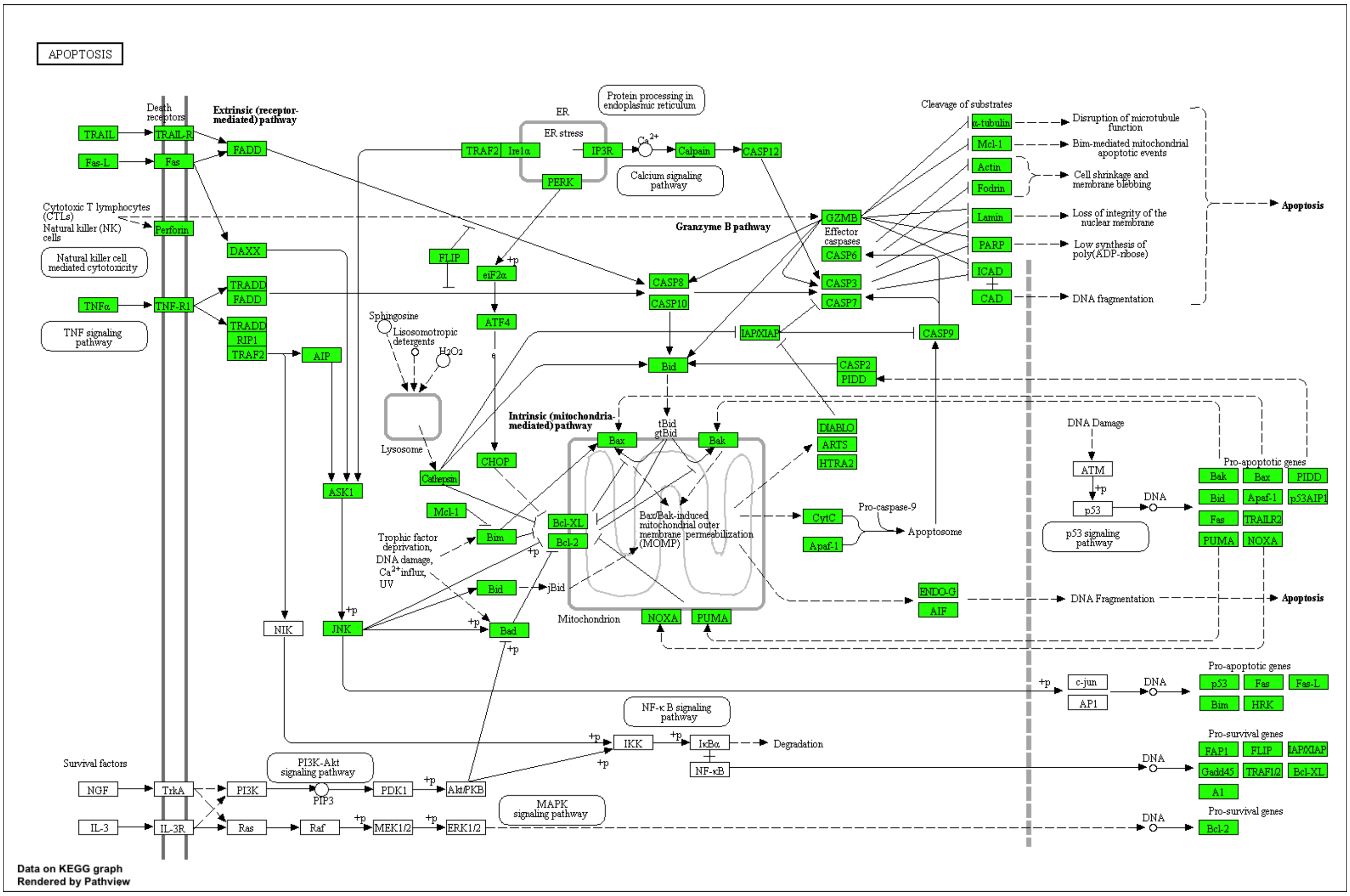

FIG. S6: **The apoptosis signaling pathway.** Based on the KEGG apoptosis signaling pathway (hsa04210), we further selected "core" pathway genes as described in the pathway definition subsection of the main text. Our analysis only used the "core" pathway genes, which are hightlighted in green. The figure was generated based on Pathview [4].

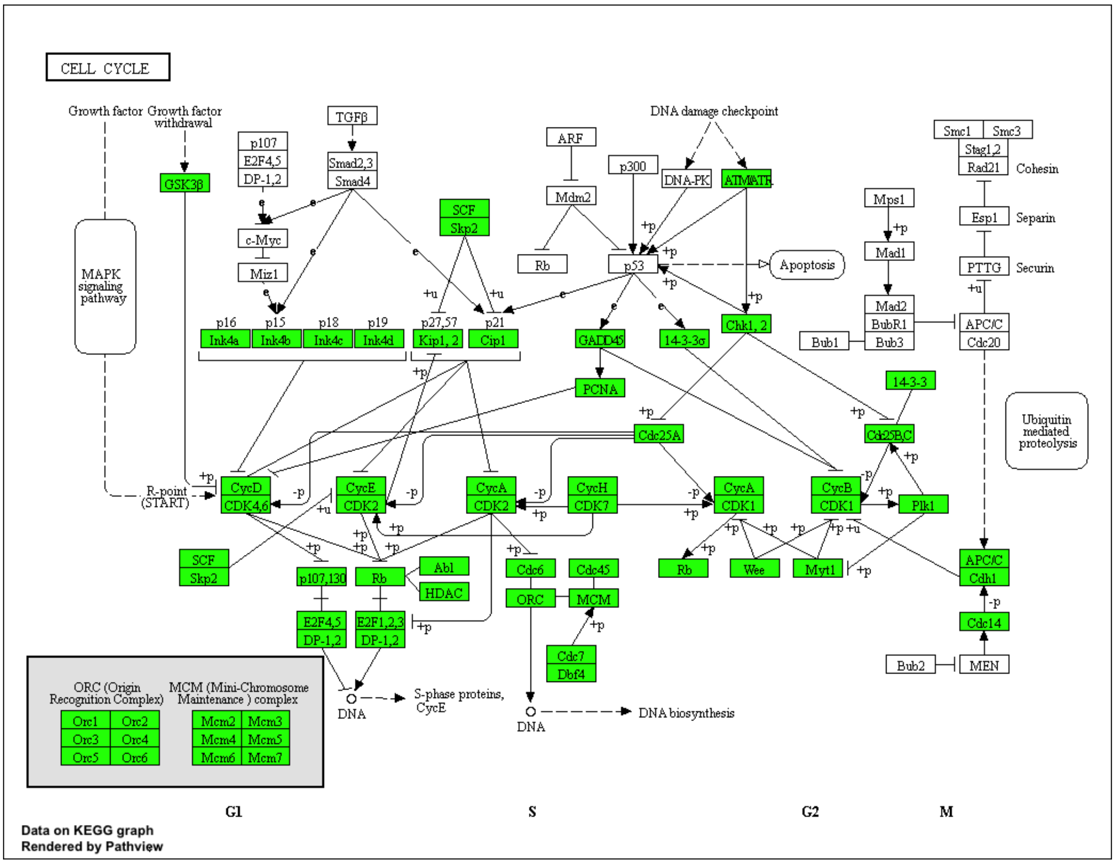

FIG. S7: **The cell cycle signaling pathway.** Based on the KEGG Cell cycle signaling pathway (hsa04110), we further selected "core" pathway genes as described in the pathway definition subsection of the main text. Our analysis only used the "core" pathway genes, which are hightlighted in green. The figure was generated based on Pathview [4].

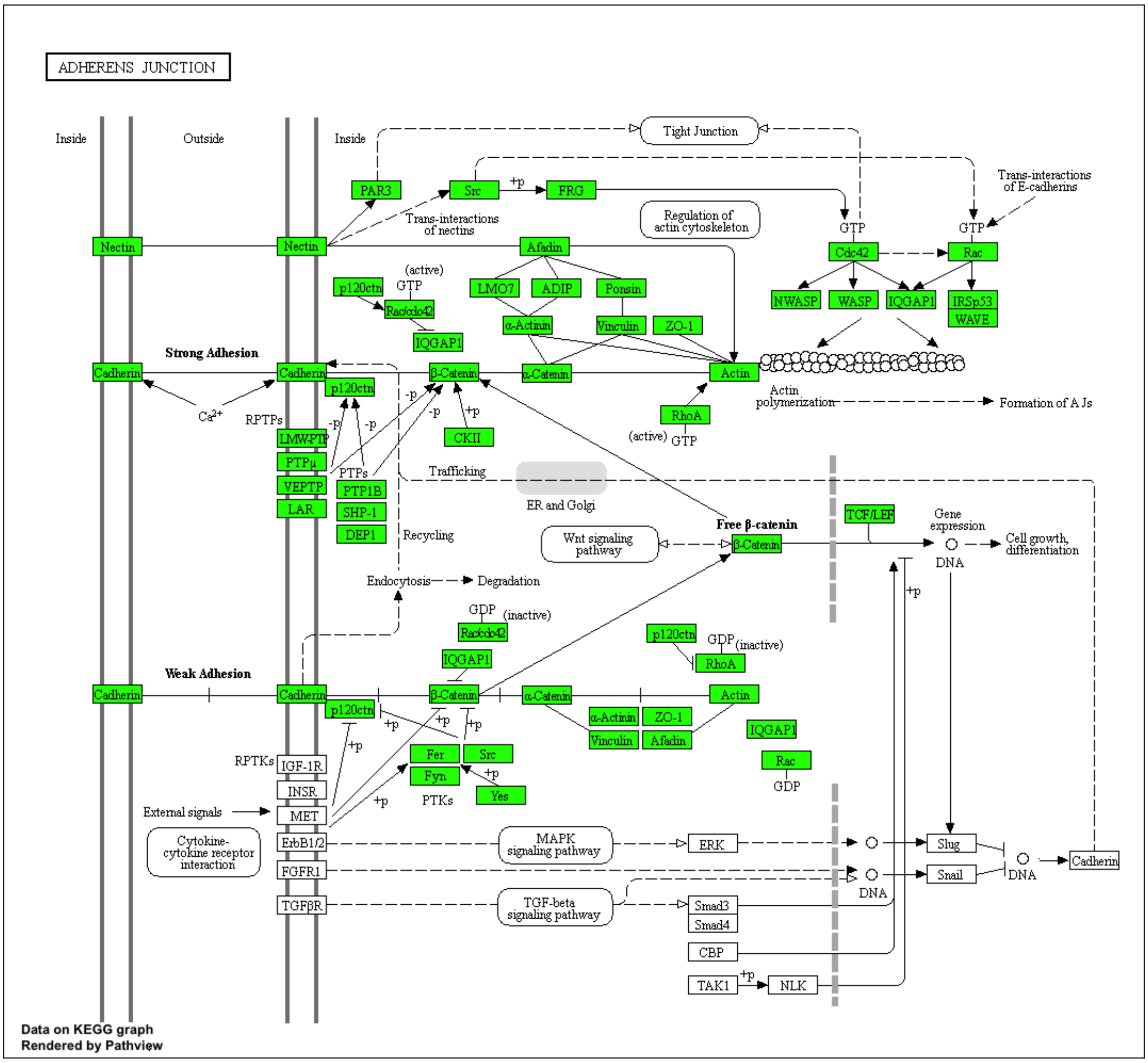

FIG. S8: **The adherens junction signaling pathway.** Based on the KEGG adherens junction signaling pathway (hsa04520), we further selected "core" pathway genes as described in the pathway definition subsection of the main text. Our analysis only used the "core" pathway genes, which are hightlighted in green. The figure was generated based on Pathview [4].

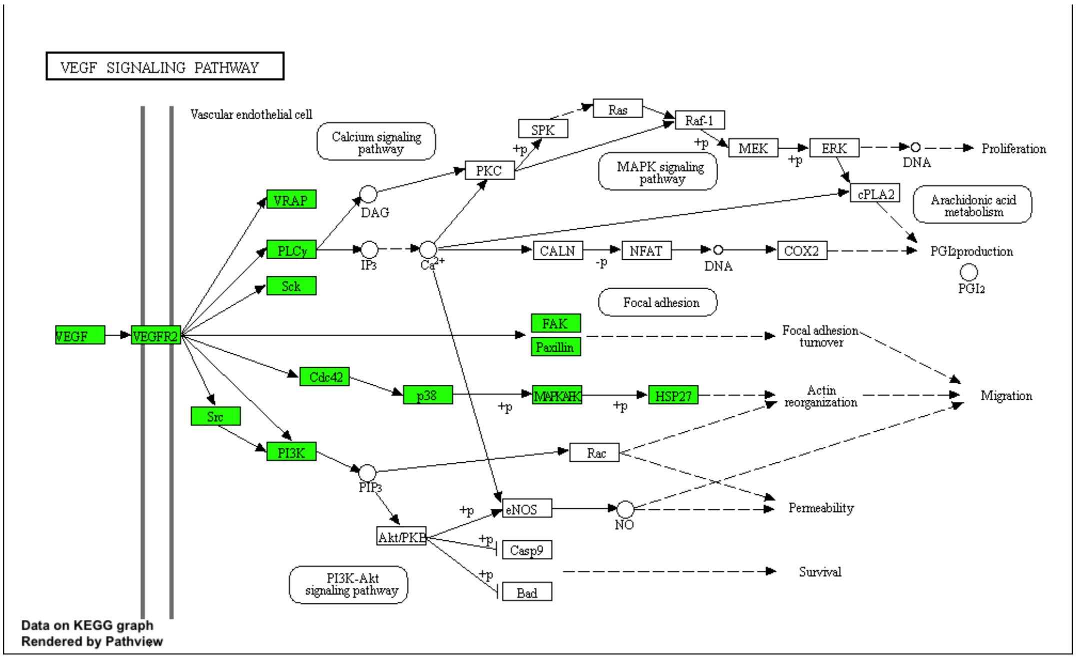

FIG. S9: **The VEGF signaling pathway.** Based on the KEGG VEGF signaling pathway (hsa04370), we further selected "core" pathway genes as described in the pathway definition subsection of the main text. Our analysis only used the "core" pathway genes, which are hightlighted in green. The figure was generated based on Pathview [4].

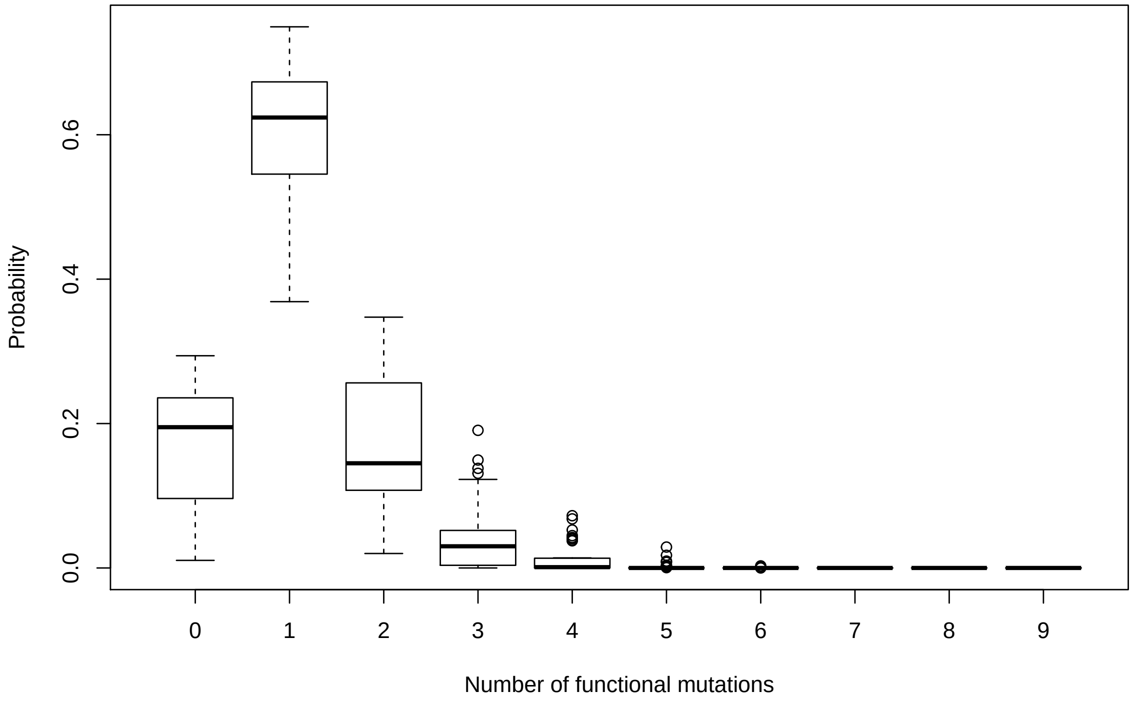

FIG. S10: **Distribution of the number of functional mutations in pairs of pathways.** For each pair of the 9 key cancer pathways, we calculated the probability for the pathway pair to contain a certain number of functional mutations. Each boxplot in the figure shows the distribution of this probability over all the 36 pairs of pathways for a given number of functional mutations. The calculation was performed using TCGA rectal cancer mutation data

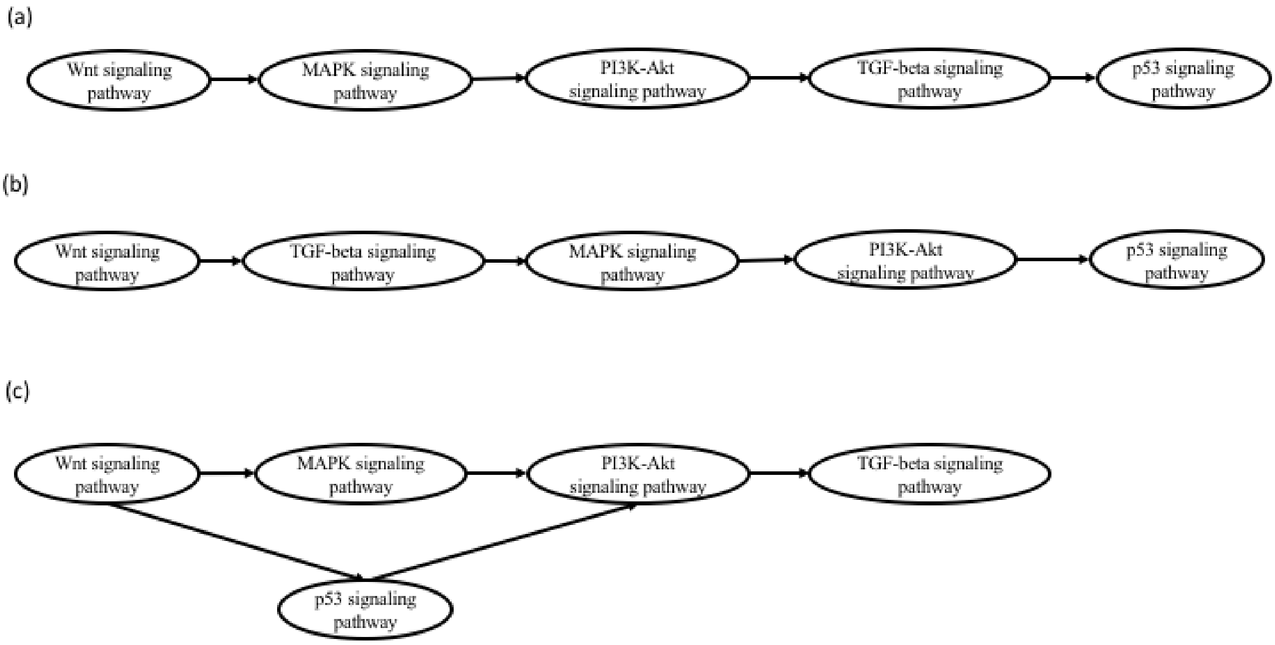

FIG. S11: **Comparison of the temporal orders of pathway alterations inferred by PATOPA with the literature.** We focus on Wnt, MAPK, PI3K, TGF-beta, and p53 signaling pathways as those are the pathways presented in the literature [3]. The figure compares temporal orders of these pathways a) reported in the literature for colorectal combined tumor; b) inferred by PATOPA using TCGA rectal cancer data; and c) inferred by PATOPA using TCGA colon cancer data. The PATOPA inferred temporal orders of WNT - MAPK - PI3K - p53 signaling pathways for rectal cancer and WNT - MAPK - PI3K - TGFbeta signaling pathways for colon cancer were the same as the known sequences of biological events in colorectal cancer. The only differences between PATOPA analysis and the literature are that TGFbeta pathway were placed before the MAPK pathway from PATOPA analysis of rectal cancer, and the p53 signaling pathway was placed before the PI3K and TGFbeta signaling pathways from PATOPA analysis of colon cancer.

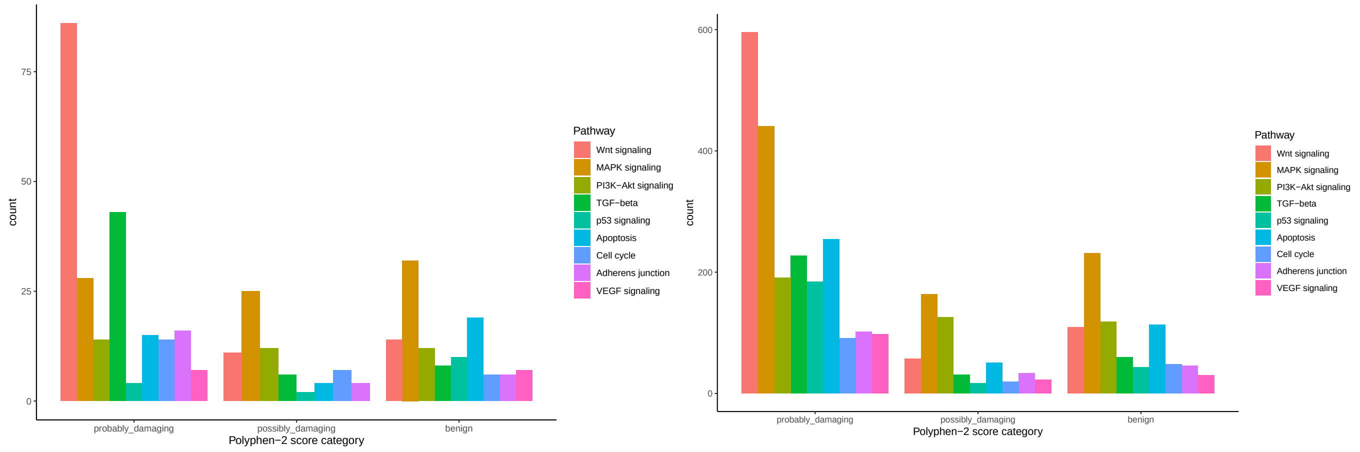

FIG. S12: **Distribution of PolyPhen-2 scores.** The PolyPhen-2 scores were categorized into probably damaging (supposed with high confidence to affect protein function or structure), possibly damaging (supposed to affect protein function or structure), or benign (most likely lacking any phenotypic effect) [1]. The categorized data were downloaded from TCGA. For each pathway, we calculated frequencies of its mutations falling into these categories. Left panel: rectal cancer; right panel: colon cancer.

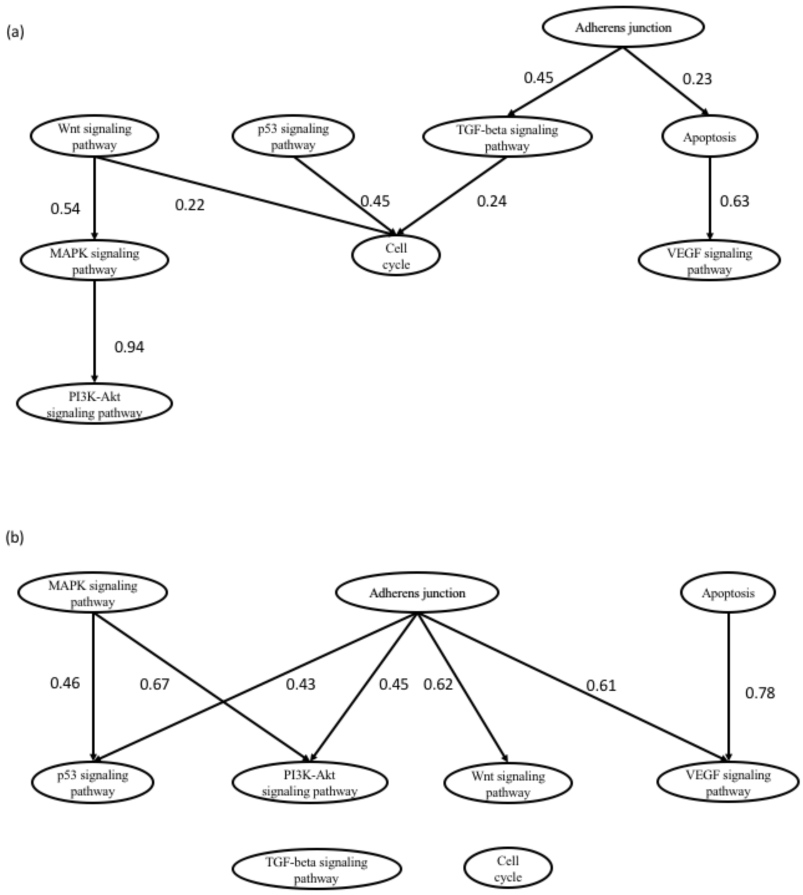

FIG. S13: **Order of pathway mutations for colorectal cancer inferred by H-CBN.** For methods comparison, we applied H-CBN [2] to TCGA rectal (a) and colon (b) cancer mutation data. We used H-CBN method with default parameter settings to get estimated progression networks, which indicate the dependency orders of pathway mutations. We performed 100 bootstraps for each cancer type and the fraction of the times that an order was validated in bootstrap was shown beside the corresponding arrow.

[1] Adzhubei, I. A., Schmidt, S., Peshkin, L., Ramensky, V. E., Gerasimova, A., Bork, P., Kondrashov, A. S., and Sunyaev, S. R. (2010). A method and server for predicting damaging missense mutations. *Nature methods*, **7**(4), 248.

[2] Gerstung, M., Nakhoul, H., and Beerenwinkel, N. (2011). Evolutionary games with affine fitness functions: applications to cancer. *Dynamic Games and Applications*, **1**(3), 370.

[3] Kuipers, E., M Grady, W., Lieberman, D., Seufferlein, T., J Sung, J., Boelens, P., H Cornelis, J., De Velde, V., and Watanabe, T. (2015). Colorectal cancer. *Nature Reviews Disease Primers*, **ePub ahead of print**.

[4] Luo, W. and Brouwer, C. (2013). Pathview: an r/bioconductor package for pathway-based data integration and visualization. *Bioinformatics*, **29**(14), 1830–1831.
